# Clinical and pathological analysis of indolent T-cell lymphoproliferative disease of the gastrointestinal tract

**DOI:** 10.3389/fimmu.2025.1530149

**Published:** 2025-05-21

**Authors:** Dan Yuan, Na Liang, Dong-Yue Wang, Jin-Jing Wang, Cong-Wei Jia

**Affiliations:** ^1^ Department of Pathology, Affiliated Hospital of Zunyi Medical University, Zunyi, China; ^2^ Department of Histology and Embryology, School of Basic Medicine, Zunyi Medical University, Zunyi, China; ^3^ Department of Pathology, Jilin Municipal People’s Hospital, Jilin, China; ^4^ Department of Pathology, Peking Union Medical College Hospital, Beijing, China

**Keywords:** Indolent T-cell lymphoproliferative disease of the gastrointestinal tract (ITLPD-GT), clinical and pathological features, abnormal positive expression of CD20, TCR monoclonal rearrangement, treatment

## Abstract

**Objective:**

This study aimed to investigate the clinicopathological features of indolent T-cell lymphoproliferative disease of the gastrointestinal tract (ITLPD-GI) and to improve its diagnostic and therapeutic approaches.

**Methods:**

A retrospective analysis was conducted on eight ITLPD-GI patients treated between January 2018 and January 2024. Clinical data, pathological features, immunophenotypes, molecular testing results, and follow-up records were reviewed.

**Results:**

Clinical characteristics: Male-to-female ratio 3:5; mean age at onset 42 years. Symptoms: Predominantly diarrhea and abdominal pain. Endoscopic findings: Erosions, multiple shallow ulcers, and small polypoid lesions. Pathological features: Histology: Atrophy of gastric/intestinal glands with diffuse infiltration of small lymphocytes (round/irregular nuclei, dense chromatin) in the lamina propria; rare mitoses; absence of angioinvasion or necrosis. Notably, two cases showed prominent plasma cell infiltration in the superficial mucosa. Immunophenotype: Pan-T-cell markers positive (5/8); CD4−/CD8+ (5/8), CD4+/CD8+ (2/8), CD4+/CD8− (1/8); aberrant CD20 expression (2/8); low Ki-67 index. TCR rearrangement: Monoclonal in all four tested cases. Treatment and prognosis: Supportive therapy (five cases): Dietary modification, immunosuppression, immunomodulation, and anti-infective agents. Symptoms resolved in one case but persisted in four. Targeted therapy (one CD20+ case): Rituximab added, with no improvement after 14 months of follow-up. Chemotherapy (two cases): Prednisone + thalidomide; one achieved significant remission at 9 months, while the other showed no response (persistent diarrhea/anxiety) at 35 months. No disease progression was observed during follow-up.

**Conclusion:**

ITLPD-GI is a rare indolent monoclonal T-cell proliferation with non-specific clinical/endoscopic features, necessitating differentiation from aggressive lymphomas to avoid misdiagnosis and overtreatment. Diagnosis relies on histomorphology, immunohistochemistry, and TCR clonality assessment (critical for atypical cases, e.g., CD20+). The majority of patients have favorable outcomes with conservative management. Enhanced clinical awareness and novel therapeutic targets warrant further exploration.

## 1 Introduction

Indolent T-cell lymphoproliferative disease of the gastrointestinal tract (ITLPD-G) is a rare subtype of intestinal T-cell lymphoma that was first characterized by Perry et al. (1) in 2013 and subsequently incorporated into the WHO (2017) classification of hematopoietic and lymphoid neoplasms. This entity follows an indolent clinical course with a favorable prognosis and rarely progresses to aggressive lymphoma. While its exact pathogenesis remains unclear, persistent antigen stimulation (e.g., from immune dysregulation or chronic infection) has been implicated (1–3). Currently, no standardized treatment guidelines exist for ITLPD-G. The disease frequently poses diagnostic challenges due to its clinical and pathological similarities to inflammatory bowel disease and aggressive T-cell lymphomas, often leading to misdiagnosis and unnecessary overtreatment. To enhance the understanding of this condition, we present a comprehensive analysis of clinicopathological features, immunophenotypic characteristics, molecular genetic alterations, and therapeutic approaches through the retrospective evaluation of eight cases and a literature review.

## 2 Materials and methods

### 2.1 Clinical data

We retrospectively analyzed eight cases of ITLPD-GI diagnosed between January 2018 and January 2024, including six in-house biopsy specimens and two consultation cases. All pathology slides were independently reviewed by two senior hematopathologists specializing in lymphoma, with the final diagnosis established according to the 2017 WHO classification criteria for hematopoietic and lymphoid neoplasms.

### 2.2 Methods

Histopathological examination: Tissue specimens were fixed in 10% neutral buffered formalin, routinely processed for paraffin embedding, and sectioned at 4 μm thickness for hematoxylin-eosin (HE) staining and light microscopic evaluation.

Immunohistochemistry (IHC): The EnVision two-step method was employed using primary antibodies against CD2, CD3, CD5, CD7, CD4, CD8, CD56, CD20, CD79α, TIA-1, granzyme B, and Ki-67 (all purchased from Zhongshan Golden Bridge Biotechnology, Beijing, China). Staining procedures followed the manufacturer’s protocols.

T-cell receptor (TCR) gene rearrangement analysis: Genomic DNA was analyzed using the BIOMED-2 multiplex PCR protocol (Europe) on an ABI 3500DX Genetic Analyzer. The assay covered TCRβ (Vβ-Jβ and Dβ-Jβ segments), TCRγ (Vγ1-11-Jγ segments), and TCRδ (Vδ-Dδ-Jδ segments). All procedures were performed in strict accordance with the manufacturer’s instructions.

## 3 Results

Detailed clinicopathological characteristics are summarized in **Table 1**.

**Table 1:** Clinical and pathological characteristics of eight cases of indolent T-cell lymphoproliferative disease of the gastrointestinal tract.

Case	Age (y)/sex	Clinical presentation	Sites of involvement	Endoscopic findings	Immunophenotype	Clonality	Therapy	Follow-up (months)
1	64/F	Physical examination revealed an increase in lymphocytes accompanied by diarrhea	Terminal ileum and colorectum	The terminal ileum and colorectal mucosa were unremarkable (**Figures 1A, B**).	CD2(+), CD3(+), CD5(+), CD7(+), CD4(−), CD8(+), Ki-67 (index 8%), CD20(弱+), CD79α(−)	Clonality not performed	Administered prednisone and thalidomide for chemotherapy	35-month follow-up: No improvement, persistent diarrhea/anxiety
2	18/F	Diarrhea with intermittent hand and foot twitching	Duodenal bulb and descending portion, terminal ileum	The duodenal bulb and descending mucosa were swollen, with edematous terminal ileal mucosa showing slightly shortened, granular villi (**Figures 1C, D**).	CD3(+), CD4(+), CD8(+), Ki-67 (index 8%), CD20(+)	TCR-clonal	Mainly based on a gluten-free diet and symptomatic supportive treatment	24-month follow-up: Diarrhea improved
3	40/F	Intermittent upper abdominal pain	Stomach, duodenum	Diffuse gastric mucosal edema appeared as multiple large nodules with focal white moss-covered ulcers, accompanied by duodenal bulb mucosal fold enlargement (**Figure 1E**).	CD2(+), CD3(+), CD5(+), CD7(+), CD4(+), CD8(−), Ki-67 (index 10%)	Clonality not performed	Symptomatic and supportive treatment	20-month follow-up: Marked symptomatic improvement (occasional vomiting), gastroscopy revealed residual ulcers in the gastric angle/antrum
4	39/M	Bloody stools	Cecum, ascending colon, transverse colon, descending colon, rectum	Discontinuous ulcers (0.5-1.0 cm) with smooth edges and white coating were observed from the cecum to the rectum, predominantly larger in the rectum (**Figures 1F–J**).	CD2(+), CD3(+), CD5(+), CD7(+), CD4(−), CD8(+), Ki-67 (index 20%)	TCR-clonal	Symptomatic supportive treatment mainly included mesalazine and thalidomide	2-month follow-up: Symptoms improved, no bloody stools
5	16/M	Abdominal pain, diarrhea with fever	Cecum, ascending colon, transverse colon, descending colon, sigmoid colon, rectum	The cecum showed scattered, patchy erosions and shallow ulcers. Colorectal mucosa (ascending to rectum) exhibited congestion and swelling with irregular white-coated ulcers, more prominent in the sigmoid colon and rectum (**Figures 1K–P**).	CD2(+), CD3(+), CD5(+), CD7(+), CD4(−), CD8(+), Ki-67 (index 15%)	TCR-clonal	Symptomatic supportive treatment mainly included mesalazine and thalidomide	12-month follow-up: Partial improvement with persistent diarrhea/low-grade fever.Colonoscopy: Multiple ulcers (slightly reduced than before)
6	52/F	Diarrhea and vomiting	Duodenum and jejunum	Duodenal villi showed shortening with scattered white, needle-like bumps. Proximal jejunal villi exhibited diffuse shortening/flattening, coarse granularity, and lymphatic dilation, and mid/distal jejunal villi exhibited mild shortening with scattered white dots and clustered lymphatic dilation (**Figures 1Q,R**).	CD2(+), CD3(+), CD5(+), CD7(+), CD4(−), CD8(+), Ki-67 (index 5%)	Clonality not performed	Administered chemotherapy with metoprolol, thalidomide, golexitinib, and prednisone	9-month follow-up: Significant improvement, normal bowel habits, weight gain
7	54/M	Repeated fatigue, diarrhea, and fever	Duodenal bulb, descending duodenum, jejunum	Mucosal elevations were noted in the duodenal bulb, descending duodenum, and jejunum.	CD3(+), CD5(+), CD7(+), CD4(−), CD8(+), Ki-67 (index 5%)	Clonality not performed	Symptomatic supportive treatment, including thalidomide	17-month follow-up: No improvement, persistent diarrhea/occasional constipation.
8	50/F	Intermittent diarrhea with abdominal distension	Ileum, rectum	The ileal and rectal mucosa showed irregularity with scattered bumps and multiple ulcers.	CD3(+), CD5(+), CD4(+), CD8(+), Ki-67 (index 1%), CD20(+), PAX-5(+)	TCR-clonal	Treated with cefotaxime and targeted drugs (i.e., rituximab)	14-month follow-up: No improvement, persistent diarrhea/fever.

### 3.1 Clinical characteristics

The cohort comprised eight patients (five women, three men) with a mean age of 42 years (median: 45 years), demonstrating a female predominance (F:M ratio 1.7:1). All patients presented with chronic diarrhea and abdominal pain, while concurrent symptoms included fever (n=3), vomiting (n=2), and hematochezia (n=1). Notably, one patient was initially identified by laboratory findings of lymphocytosis (WBC 12-15×10^9^/L, lymphocyte percentage 80%) before developing diarrhea.

### 3.2 Endoscopic findings

**Figure 1:**
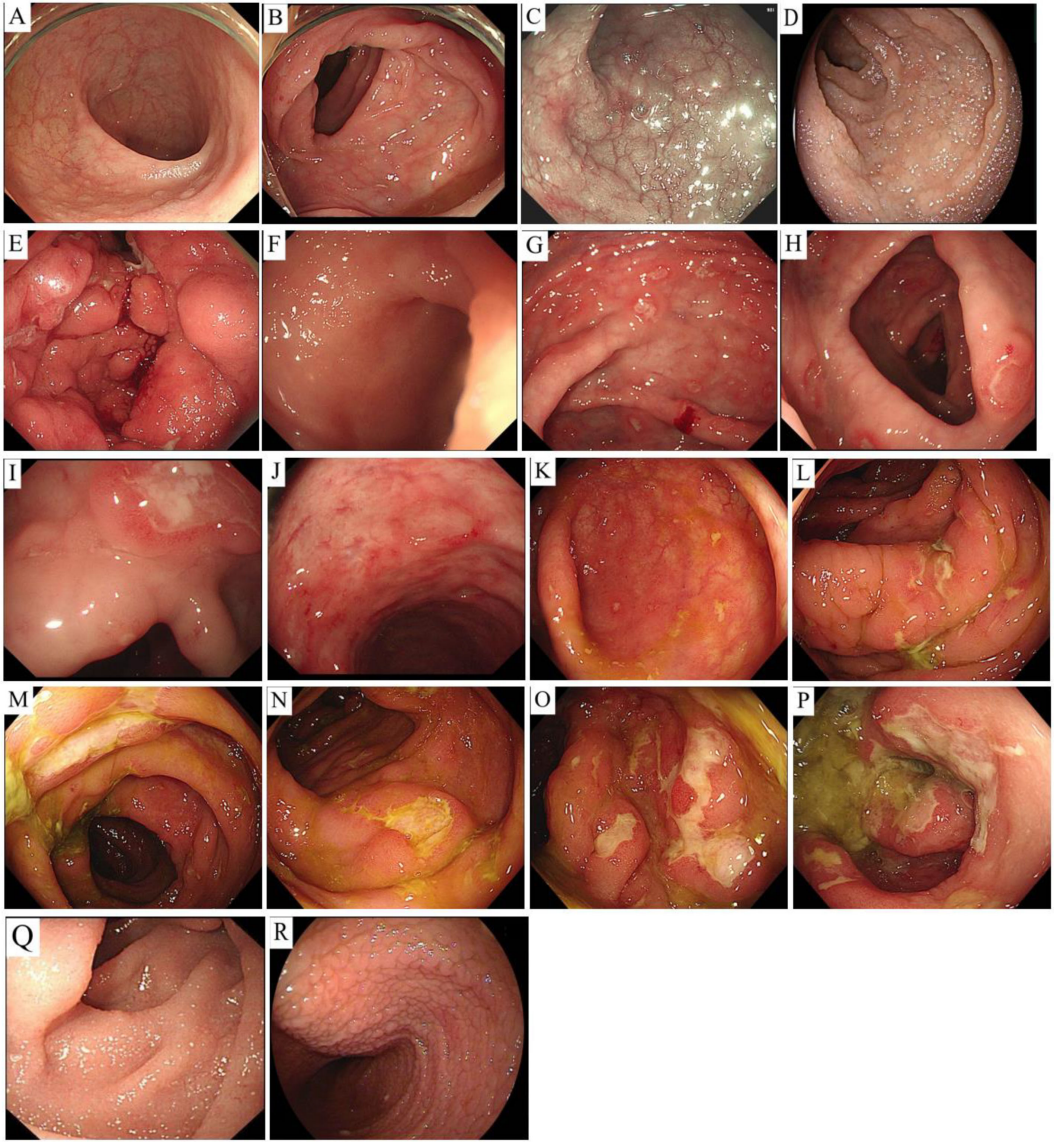
Endoscopic findings. Case 1: Terminal ileum and colorectal mucosa are unremarkable **(A, B)**. Case 2: Duodenal bulb/descending mucosa swelling **(C)**; terminal ileum edema with granular, shortened villi **(D)**. Case 3: Diffuse gastric edema with nodular changes and focal white-coated ulcers **(E)**. Case 4: Non-continuous ulcers were observed in the cecum **(F)**, ascending colon **(G)**, transverse colon **(H)**, descending colon **(I)**, and rectum **(J)**, with smooth edges and a white coating, ranging in size from 0.5-1.0 cm. Larger lesions were more common in the rectum **(F-J)**. Case 5: Scattered patchy erosions and shallow ulcers were observed in the cecum **(K)**, while the mucosa of the ascending colon **(L)**, transverse colon **(M)**, descending colon **(N)**, sigmoid colon **(O)**, and rectum **(P)** were congested and swollen. Multiple irregular, patchy ulcers were scattered and covered with a white coating, with larger and more obvious ulcers in the sigmoid colon and rectum **(K–P)**. Case 6: Duodenal villi were shortened with white and white needle-like protrusions **(Q)**; proximal jejunal villi were diffusely flattened/granular with lymphatic dilation, and mid/distal jejunal villi were mildly shortened with white dots **(R)**.

Endoscopic evaluations revealed erosive changes with multiple ulcers and mucosal hyperemia/edema (n=3); multiple small protrusions with diffuse villous shortening, flattening, and granular appearance (n=3); combined ulcerative and protrusive lesions (n=1); and normal endoscopic findings (n=1). All cases had multi-site gastrointestinal involvement, with six cases having small intestine involvement (n=6), including two cases with concurrent large intestine involvement and one with additional gastric involvement. Two cases had isolated large intestine involvement (**Figure 1**).

### 3.3 Treatment outcomes

Therapeutic interventions yielded variable responses:

1. Supportive care group (n=5; dietary modification, immunomodulation, anti-infectives): This treatment led to partial symptom improvement with residual diarrhea/vomiting/fever in four cases and complete resolution in one.
2. Targeted therapy (n=1; CD20+ case with rituximab): This treatment led to no clinical improvement after 14 months of follow-up (persistent diarrhea/fever).
3. Chemotherapy group (n=2; prednisone + thalidomide): This treatment led to significant improvement at 9 months of follow-up in one case and treatment failure at 35 months of follow-up (ongoing diarrhea/anxiety) in another.

### 3.4 Histopathological features

Gross examination: All specimens consisted of 2–5 endoscopic biopsy fragments (diameter: 2–3 mm).

**Figure 2:**
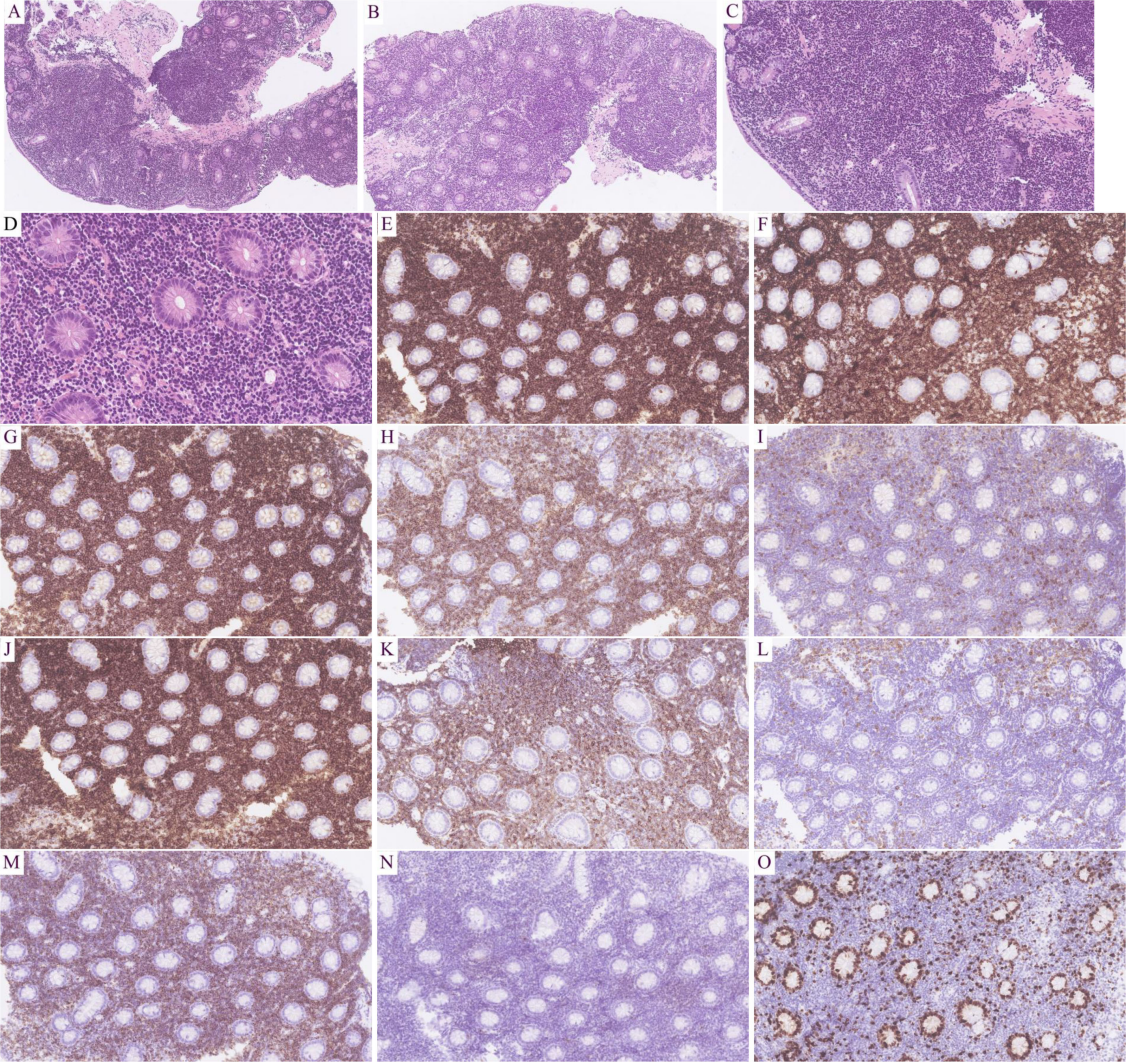
Results of hematoxylin-eosin (HE) staining and immunohistochemistry (IHC) assays. HE: Intestinal gland atrophy with diffuse lymphoid infiltration of the lamina propria (**A**, **B**; 100×).Uniform small cells: scant cytoplasm, round/irregular nuclei, dense chromatin, inconspicuous nucleoli, rare mitosis (**C**, 200×; **D**, 400×).IHC: T-cell markers: CD2+(**E**; 200×), CD3+(**F**; 200×), CD5+(**G**; 200×), CD7+(**H**; 200×); Subset: CD4−(**I**; 200×), CD8+ (**J**; 200×); Aberrant: CD20+ (**K**; 200×), CD79α− (**L**; 200×); Cytotoxic: TIA1+ (**M**; 200×), Granzyme B− (**N**; 200×); Proliferation: Ki-67 ~10% (**O**; 200×).

**Figure 3:**
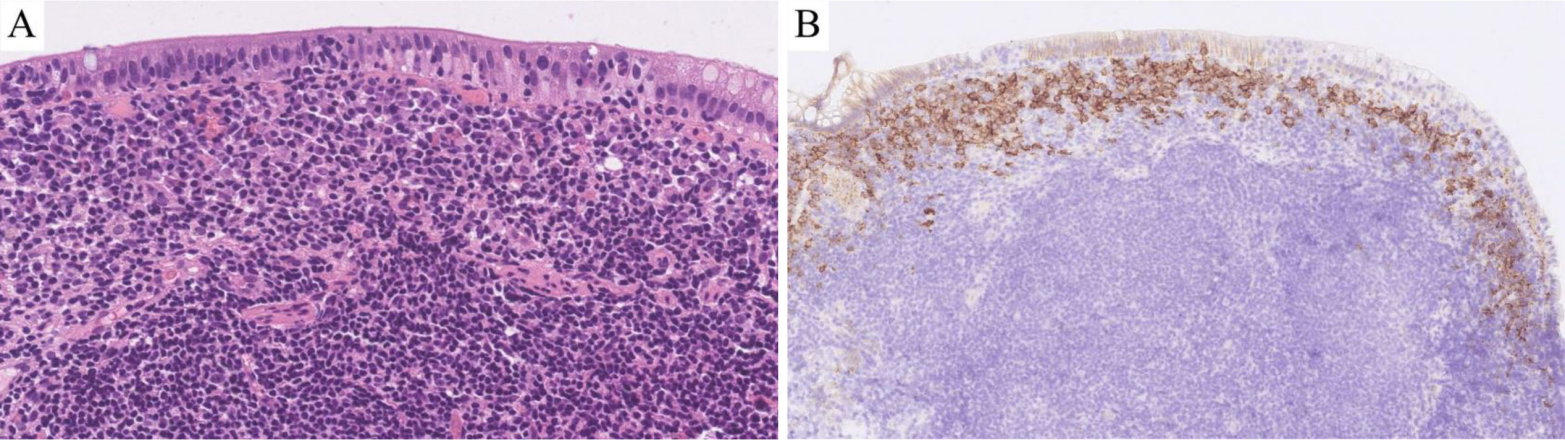
Results of hematoxylin-eosin (HE) staining and immunohistochemistry (IHC) assays. **HE:** There was a large amount of plasma cell infiltration in the superficial mucosa (**A**; HE 400×).**IHC:** There was a large amount of CD138-positive plasma cell infiltration in the superficial mucosa (**B**; IHC 200×).

Microscopic findings: All eight cases exhibited identical histological features. Glandular architecture: Atrophy of gastric/intestinal mucosal glands. Infiltrate pattern: Dense, monotonous lymphoid infiltration confined to the lamina propria (**Figures 2A, B**). Cytomorphology: Small lymphocytes with scant cytoplasm; round to mildly irregular nuclei with condensed chromatin; inconspicuous nucleoli; rare mitotic figures or apoptosis. Key negative findings: No angioinvasion, necrosis, or lymphoepithelial lesions; infiltration strictly limited to the mucosal layer (**Figures 2C, D**). Additional observations: Mild scattered plasma cells and eosinophils in the superficial mucosa (six cases); prominent plasma cell infiltration in the superficial mucosa (two cases) (**Figure 3A**).

### 3.5 Immunophenotypic and TCR gene rearrangement characteristics

T-cell markers: Pan-T markers [CD2(**Figure 2E**), CD3 (**Figure 2F**), CD5 (**Figure 2G**), CD7 (**Figure 2H**)] were positive in five cases. One case with limited panel: CD3+ only. Two consultation cases: Case 1: CD3+/CD5+/CD7+; Case 2: CD3+/CD5+. CD4/CD8 subsets: CD4-/CD8+ (five cases, **Figures 2I, J**): TIA1+ (**Figure 2M**) /Granzyme B- (**Figure 2N**) (three cases), TIA1+/Granzyme B+ (one case), not tested (one case); CD4+/CD8+ (two cases): TIA1+/Granzyme B- (one case), not tested (one case); CD4+/CD8- (one case, not tested). Aberrant markers: CD20+ (two cases, **Figure 2K**), one co-expressed PAX5 (but CD79α-, **Figure 2L**). CD56- in all cases. Proliferation index: Low Ki-67 (<15%, **Figure 2O**). Plasma cell infiltration: CD138+ plasma cell clusters in the superficial mucosa (two cases, **Figure 3B**). TCR gene rearrangement: Monoclonal TCR rearrangement confirmed in 4/4 tested cases; four cases not tested (see **Table 1**).

## 4 Discussion and conclusion

### 4.1 Clinical characteristics

ITLPD-GI remains exceptionally rare, with less than 80 cases reported in the literature as of 2021, and a total of less than 90 documented cases (4–9). Our cohort of eight patients (F:M ratio 1.7:1, mean age 42 years) showed a slight female predominance compared to previous reports (M:F ≈1.5:1) (4), which may reflect the limited sample size.

### 4.2 Etiology and pathogenesis

The exact etiology is unknown, but persistent antigen stimulation—potentially driven by immune-mediated or infectious conditions (e.g., IBD, viral infections, autoimmune disorders, or Helicobacter pylori infection)—is hypothesized to play a role (1–3). In our series, three cases arose from long-standing inflammatory bowel disease (IBD; Crohn’s disease/ulcerative colitis), supporting the theory of chronic immune stimulation leading to monoclonal T-cell proliferation (9). Notably, TNF-α inhibitors have been implicated in triggering ITLPD-GI, with documented regression upon discontinuation (10).

### 4.3 Clinical manifestations

Typical symptoms: Diarrhea, abdominal pain, fever, vomiting, and hematochezia, consistent with previous reports (4–9).

Novel findings: One case presented with peripheral lymphocytosis (WBC 12–15×10^9^/L, LY% 80%), possibly linked to sustained antigen exposure, although further studies are needed.

Other rare manifestations: Our cohort included tetany while the literature also describes refractory oral ulcers, anal fistulae (8, 9, 11), metabolic disturbances (hypocalcemia/hypokalemia/hypomagnesemia/hypozincemia), and neurological symptoms (paresthesia, confusion) (1). Asymptomatic cases are occasionally detected incidentally during endoscopy or lymph node biopsy (12).

### 4.4 Disease distribution

Gastrointestinal involvement: All cases exhibited multi-site involvement (six cases: small intestine ± large intestine/stomach; two cases: large intestine only), aligning with published data (4–9, 13). Lesions predominantly affected the small bowel/colon, with sporadic gastric/esophageal involvement, typically showing discontinuous focal distribution.

Extraintestinal spread: In patients with this condition, rare cases involve the liver, bone marrow, peripheral blood, or lungs (4, 6, 9, 14, 15), with isolated reports of vitreoretinal infiltration (16).

### 4.5 Endoscopic features

None of the eight cases displayed pathognomonic endoscopic findings. Observations included erosions, shallow ulcers, mucosal hyperemia/edema, and villous shortening (one case appeared normal), mimicking IBD. Notably, mass formation or perforation is uncommon (4–9, 13).

### 4.6 Pathological features

#### 4.6.1 Histomorphology

The diagnosis of ITLPD-G relies on histopathological examination, as both clinical and endoscopic findings lack specificity.

Characteristic features: Mucosal glandular atrophy with dense, monotonous infiltration of small lymphocytes in the lamina propria. Cytomorphology: Round to mildly irregular nuclei, condensed chromatin, inconspicuous nucleoli. Low proliferative activity: Rare apoptosis or mitotic figures. Absence of angioinvasion, necrosis, or lymphoepithelial lesions. Superficial epithelium may show erosions.

Depth of infiltration: Typically confined to the lamina propria, with occasional extension to the muscularis mucosae/submucosa. Transmural involvement is exceedingly rare (only 1 reported case by Huang et al. (7)).

Microenvironment: Six of eight cases exhibited mild plasma cell and eosinophil infiltration in the superficial mucosa. Two of eight cases showed prominent plasma cell infiltration. This finding may serve as a diagnostic clue or prognostic indicator, although larger studies are needed for validation. Lucioni et al. (17) reported that dense superficial plasma cell infiltration in ITLPD-G may help differentiate it from other GI T-cell lymphomas, suggesting microenvironmental immune cells (e.g., plasma cells) as potential diagnostic markers.

#### 4.6.2 Immunophenotypic and molecular characteristics

T-cell markers: The majority of cases express pan-T markers (CD2/CD3/CD5/CD7), though partial loss may occur. Predominant subsets: CD4+/CD8- or CD4-/CD8+, with fewer CD4+/CD8+ or CD4-/CD8- cases (4, 9, 12, 13, 15). Our cohort: five cases expressed full T-cell markers. CD4-/CD8+ subset (n=5): three cases TIA1+/Granzyme B-; one case TIA1+/Granzyme B+; one case not tested. CD4+/CD8- (n=1): Not tested.CD4+/CD8+ (n=2): one case TIA1+/Granzyme B-; one case not tested. These results align with previous reports, wherein CD4−/CD8+ cases typically express TIA-1 but rarely Granzyme B, while CD4+/CD8− cases are usually negative for both markers (9, 18). There is a consistently low Ki-67 index.

Aberrant CD20 expression: Two cases demonstrated CD20 positivity, including one case with concurrent PAX5 expression. This is a potential diagnostic pitfall that may lead to misclassification as B-cell lymphoma. In such scenarios, TCR gene rearrangement analysis serves as a crucial confirmatory test. The underlying mechanism may involve either neoplastic transformation of specific T-cell subsets or aberrant activation-induced phenotypic alterations in T lymphocytes (14, 19).

Molecular findings: Monoclonal TCR rearrangement was diagnostic, as 4/4 tested cases were positive (including one CD20+ case); four cases were not tested.

T-helper cell polarization: CD4+ or CD4+/CD8+ cases may show Th1/Th2 hybrid phenotypes; CD8+ or CD4-/CD8- cases often exhibit Th2 polarization, and some demonstrate lineage plasticity (Soderquist et al. (12)).

### 4.7 Molecular genetic characteristics

#### 4.7.1 Key findings

Approximately 80% of ITLPD-GI cases harbor pathogenic or likely pathogenic genetic alterations (12), predominantly affecting the following pathways.

##### 4.7.1.1 JAK-STAT pathway abnormalities

STAT3-JAK2 fusions were highly prevalent in CD4+ cases (4/5 cases, Sharma et al. (20)) and may drive oncogenesis through STAT5 phosphorylation (Hu et al. (21)). STAT family phosphorylation was common in CD4+/CD8+ and double-negative cases (STAT1/2/5 phosphorylation; Soderquist et al. (12)).

##### 4.7.1.2 Epigenetic regulator mutations

There were recurrent mutations in TET2, DNMT3A, and KMT2D (4/10 cases (12)) and potential early events in lymphomagenesis (22). Co-occurring alterations included CDKN2A missense mutations and TNFAIP3 nonsense mutations (12).

##### 4.7.1.3 CD8+ case signature

In total, 50% of cases exhibit chromosomal structural alterations in the IL-2 gene 3’UTR region (12). IDH1/2 or SETD2 mutations were notably absent (12, 23).

##### 4.7.1.4 Transformation-associated mutations

TP53 and POLE mutations were observed in cases progressing to aggressive lymphoma (12).

### 4.8 Differential diagnoses of ITLPD-GI

#### 4.8.1 Diagnostic challenges

ITLPD-GI poses significant diagnostic difficulties due to its rarity and non-specific clinical manifestations, frequently leading to misdiagnosis as IBD or aggressive T-cell lymphomas. Accurate diagnosis requires a comprehensive evaluation incorporating histopathology, immunohistochemistry, and molecular testing (**Table 2**).

**Table 2:** Key differential diagnoses and distinguishing features.

Disease	Key distinguishing features	ITLPD-GI characteristics
MEITL	- Ulcerative masses with intestinal obstruction/perforation - Transmural infiltration, high mitotic rate, intraepithelial lesions, CD56+, high Ki67 - SETD2/H3K36me3 loss (24)	- Mucosa-limited infiltration, low mitotic rate - CD56-, low Ki67, intact SETD2
EATL	- Common in Western populations; associated with celiac disease - Transmural infiltration, marked atypia, CD30+/TIA1+, high Ki67 - JAK1/STAT3 mutations (25)	- Small monotonous cells, mucosa-confined, low Ki67, CD30- - No celiac disease association
NK/T-cell Lymphoma	- Necrosis, angioinvasion, EBER+ - CD56+, high Ki67	- No necrosis/angioinvasion, EBER- - Low Ki67
EBV+ T-LPD	- Systemic multi-organ involvement, EBER+ (26)	- Gastrointestinal-restricted, EBER-
PTCL (Peripheral T-cell Lymphoma)	- High-grade cytologic atypia, high Ki67	- Bland cytomorphology, low Ki67
MALToma (B-cell)	- CD20+/PAX5+, polyclonal TCR	- Rare CD20+ (aberrant), monoclonal TCR
IBD	- Crypt abscesses/granulomas, polyclonal TCR	- No crypt abscesses, monoclonal TCR

MEITL, monomorphic epitheliotropic intestinal T-cell lymphoma; EATL, enteropathy-associated T-cell lymphoma; EBV+T-LPD, EBV-positive T-cell lymphoproliferative disease; MALToma, extranodal marginal zone lymphoma of mucosa-associated lymphoid tissue; IBD, inflammatory bowel disease.

#### 4.8.2 Key diagnostic considerations

##### 4.8.2.1 Endoscopic/histopathological features

ITLPD-GI: Superficial mucosal involvement without deep ulceration/perforation; small, monotonous lymphocytes with bland cytomorphology; rare mitotic figures (<5/10 HPF); absence of necrosis/angioinvasion; low proliferative index (Ki67 <15%).

Aggressive lymphomas: Transmural infiltration; marked cytologic atypia; frequent mitoses (>10/10 HPF); common necrosis/angiodestruction; high Ki67 (>50%).

##### 4.8.2.2 Molecular diagnostics

Monoclonal TCR rearrangement serves as the diagnostic gold standard and is particularly crucial for atypical presentations (e.g., CD20+ cases).

##### 4.8.2.3 Clinical caveats

Diagnostic pitfalls in our series included two cases that were initially misdiagnosed (as MALToma and EATL/MEITL). These were corrected through repeat biopsy with TCR clonality assessment. Thus, we recommend maintaining high suspicion for ITLPD-GI in cases with chronic GI symptoms refractory to IBD therapy, with discordant histological and immunophenotypic findings.

### 4.9 Treatment and prognosis

Currently, there is no consensus on the treatment of ITLPD-GI, with “watchful waiting” being the primary recommended strategy (4, 9). Conventional chemotherapy typically shows limited efficacy (4), although some alternative therapies have demonstrated certain therapeutic effects. Low-dose radiotherapy (30 Gy/20 fractions) successfully treated a gastric case with 1-year recurrence-free survival (27). Furthermore, corticosteroids or anti-CD52 monoclonal antibodies can improve symptoms (3, 5). Metronomic chemotherapy (prednisone + cyclophosphamide + thalidomide) achieved 1-year clinical remission in patients with severe symptoms (5).

In our case series, of the two patients treated with prednisone plus thalidomide, one showed improvement after 9 months, while the other still experienced diarrhea and anxiety after 35 months, suggesting the need for psychological intervention. Of the five patients receiving symptomatic treatment, four showed varying degrees of symptom improvement (although with residual diarrhea, vomiting, or fever), while one achieved complete resolution. Notably, one CD20-positive patient with ITLPD-GI showed no response to rituximab after 14 months of treatment, indicating that its efficacy in T-cell lymphomas requires further validation (19).

Although ITLPD-GI typically follows an indolent course (median survival >10 years) (4), there is a risk of progression. ITLPD-GI can transform into aggressive T-cell lymphomas (e.g., ALCL), often with hepatic/bone marrow involvement and an extremely poor prognosis (2, 11, 20). The transformation mechanism may correlate with the CD4-positive phenotype (accounting for 60% of progressive cases) or genetic mutations, while CD8-positive cases may have a better prognosis (11, 13, 20). Additionally, ITLPD-GI may coexist with other lymphomas (e.g., diffuse large B-cell lymphoma, Hodgkin’s lymphoma) (28, 29) or autoimmune diseases (this series is the first to report co-occurrence with Castleman disease and celiac disease, the latter improved with a gluten-free diet). Notably, one case initially misdiagnosed as EATL showed a transient response to a PD-1 inhibitor before progressing to confirmed ITLPD-GI, highlighting the diagnostic complexity. However, this study has limitations. Given the rarity of ITLPD-GI, only eight cases were included. Future research should expand the cohort and investigate molecular mechanisms to refine treatment strategies.

In conclusion, ITLPD-GI is a rare indolent monoclonal T-cell proliferative disorder with non-specific clinical and endoscopic manifestations, requiring differentiation from aggressive T-cell lymphomas (e.g., MEITL, EATL) and IBD to avoid misdiagnosis and unnecessary treatment. Diagnosis primarily relies on histopathology (mucosa-limited small lymphocyte infiltration) and immunohistochemistry, while TCR gene monoclonal rearrangement testing is crucial for confirmation in cases with atypical immunophenotypes (e.g., aberrant CD20 expression). The disease typically follows an indolent course, potentially associated with chronic antigen stimulation (e.g., IBD, infection), with the majority of patients having a favorable prognosis, and “watchful waiting” being recommended. However, a minority of cases may progress to aggressive T-cell lymphoma with a significantly worse prognosis, although the exact transformation mechanisms remain unclear. Therefore, enhancing awareness among clinicians and pathologists is critical, and future research should further explore the molecular mechanisms to optimize treatment strategies.

## Data Availability

The original contributions presented in the study are included in the article/supplementary material. Further inquiries can be directed to the corresponding author.
